# A Novel Suture Training System for Open Surgery Replicating Procedures Performed by Experts Using Augmented Reality

**DOI:** 10.1007/s10916-021-01735-6

**Published:** 2021-04-07

**Authors:** Yuri Nagayo, Toki Saito, Hiroshi Oyama

**Affiliations:** Department of Clinical Information Engineering, Graduate School of Medicine, University of Tokyo, 7-3-1 Hongo, Bunkyo-Ku, Tokyo, 113-0033 Japan

**Keywords:** Surgical training, Open surgery, Augmented reality, Simulation-based training, Motion capture, Replication

## Abstract

The surgical education environment has been changing significantly due to restricted work hours, limited resources, and increasing public concern for safety and quality, leading to the evolution of simulation-based training in surgery. Of the various simulators, low-fidelity simulators are widely used to practice surgical skills such as sutures because they are portable, inexpensive, and easy to use without requiring complicated settings. However, since low-fidelity simulators do not offer any teaching information, trainees do self-practice with them, referring to textbooks or videos, which are insufficient to learn open surgical procedures. This study aimed to develop a new suture training system for open surgery that provides trainees with the three-dimensional information of exemplary procedures performed by experts and allows them to observe and imitate the procedures during self-practice. The proposed system consists of a motion capture system of surgical instruments and a three-dimensional replication system of captured procedures on the surgical field. Motion capture of surgical instruments was achieved inexpensively by using cylindrical augmented reality (AR) markers, and replication of captured procedures was realized by visualizing them three-dimensionally at the same position and orientation as captured, using an AR device. For subcuticular interrupted suture, it was confirmed that the proposed system enabled users to observe experts’ procedures from any angle and imitate them by manipulating the actual surgical instruments during self-practice. We expect that this training system will contribute to developing a novel surgical training method that enables trainees to learn surgical skills by themselves in the absence of experts.

## Introduction

Surgeons traditionally learned surgical skills by the “see one, do one, teach one” method, developed by Dr. William Halsted [1–4]. However, it is challenging to acquire surgical skills by this method alone because of the lack of resident training time, limited educational resources at each surgical center, and increased patient demands for safety and quality [1–6].

Simulation-based surgical skill training is an educational approach that can provide trainees the opportunity to experience a given task or situation in a safe environment. It provides standardized and reproducible contents to practice surgical skills, allowing trainees to practice them repeatedly until they acquire the skills [4, 5, 7]; its importance in resident education is increasingly recognized, and previous studies have demonstrated that it improved surgical skills [3, 4, 8, 9].

There are different types and complexities of simulators for surgical training [10]. Among these, low-fidelity simulators, such as skin pads, include limited functionality to meet selected requirements to improve surgical techniques, and suture is one of the essential skills trainees can practice using them. The low-fidelity simulators are generally portable, inexpensive, and easy to use, requiring no special equipment or maintenance [9]. On the other hand, when trainees use such a simulator, since the simulator does not give instructions, they need to ask a skilled surgeon (expert) for instruction [10, 11]. However, because experts’ availability is limited due to time constraints and economic issues, trainees must do self-practice with low-fidelity simulators, referring to textbooks or videos [11]. Books and videos provide instructions on procedures, but to access the information, trainees must stop practicing. In addition, most procedural information is visualized in two dimensions, and trainees cannot see the procedures in books or videos from their desired angle, because of the fixed point of view of images or videos. These features are more disadvantageous when learning open surgery than endoscopic surgery because, in endoscopic surgery, all surgeons see the same two-dimensional videos captured from an endoscope, whereas in open surgery, each surgeon perceives the procedures three-dimensionally with his or her own eyes, changing the point of view to see them from the preferred direction. Furthermore, the motion of surgical instruments in open surgery has more degrees of freedom (DOF) than in endoscopic surgery because surgeons manipulate them freely with both hands [12, 13]. Therefore, a new open surgical procedure training system is desired, allowing trainees to practice while viewing the procedures in three dimensions from the desired angle, even without an instructor.

Augmented reality (AR) is a technology that expands our physical world by overlaying digital information onto it. This technology allows users to interact with the information in the real world, and AR in medical education is gathering attention because users do not have to pause or take their eyes off the surgical field when accessing the information while practicing [2, 14, 15]. Within healthcare, AR applications have been proposed, including training in laparoscopic surgery, a navigation tool during surgeries, and a therapeutic tool for treating patients [16, 17].

However, there have been no reports of an AR system that allows trainees to observe the open surgical procedures from any angle during training.

The present study aimed to build an AR system that trainees observe and imitate exemplary procedures during practice without an instructor. This study proposes a novel suture training system for open surgery that provides three-dimensional (3D) information of experts’ procedures by combining AR technology with a low-fidelity simulator.

## Methods

### System design

The proposed system allows users to observe and imitate experts’ procedures by replicating and visualizing them on the surgical field. To implement this system, it was decided to replicate the motion of surgical instruments. Thus, this system consists of a motion capture system for surgical instruments and a 3D replication system of captured motion on the surgical field.

Subcuticular interrupted suture was chosen as a target procedure because it is one of the problematic skills trainees must acquire early in their training. AR markers were used to track surgical instruments and the surgical field in the motion capture system. HoloLens 2 (Microsoft, Inc., Bellevue, WA) was used to replicate the procedures by displaying 3D computer graphics (3D-CG) models of surgical instruments as holograms on the surgical field. The system was developed using Vuforia Engine (PTC, Inc., Boston, MA), Unity (Unity Technologies, San Francisco, CA), and MRTK (Microsoft, Inc.).

### Motion capture system for surgical instruments

**Fig. 1:**
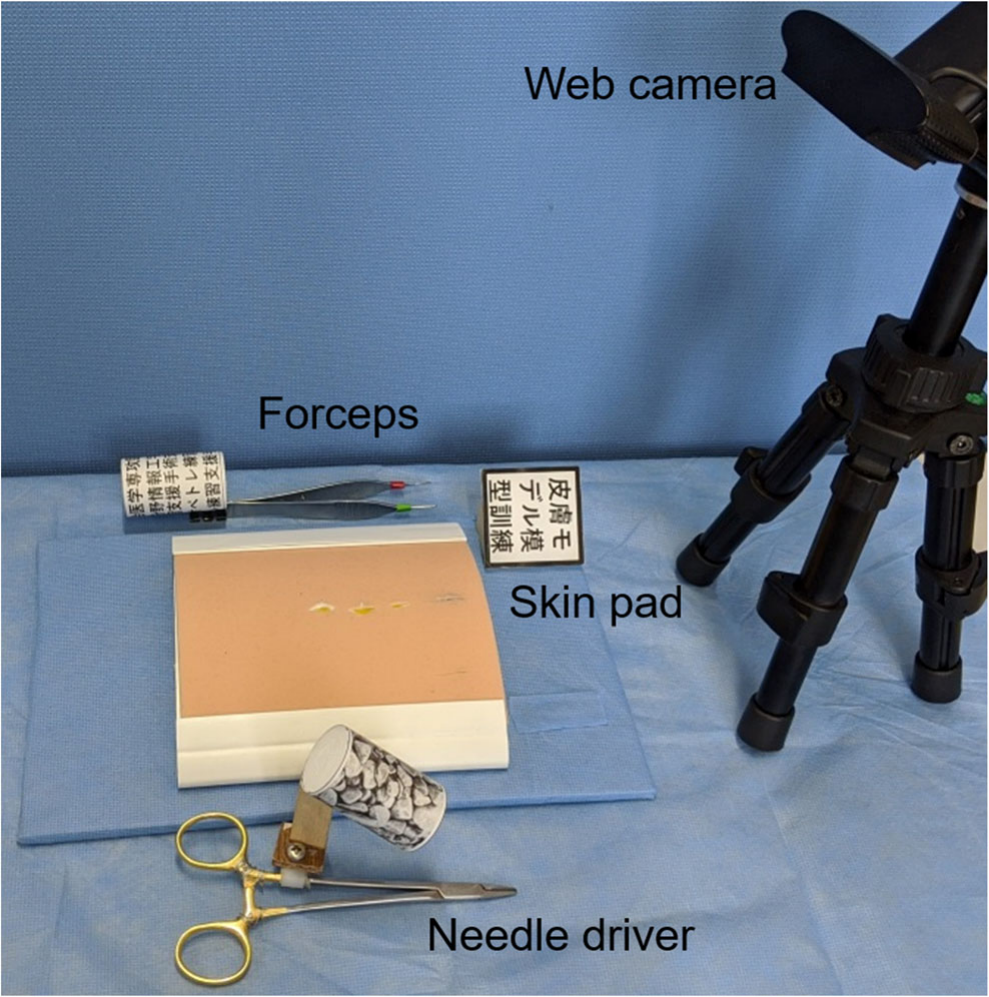
Setup of a motion capture system for subcuticular interrupted suture. The low-cost, portable setup includes surgical instruments with AR markers, a skin pad with an AR marker, and a web camera

The setup of the motion capture system is shown in Fig. 1. All sutures were performed on a skin pad (Limbs & Things, Bristol, UK) using a 16.5-cm Olsen-Hegar needle driver, an Adson forceps, and 4–0 PDS (Ethicon Inc., Bridgewater, NJ). A C920 HD Pro Webcam (Logicool, Lausanne, Switzerland) was set approximately 300 mm above the skin pad to track the AR markers. Two AR markers were attached to each surgical instrument. A 50-mm-square AR marker was attached next to the skin pad as a reference marker on the surgical field.

**Fig. 2:**
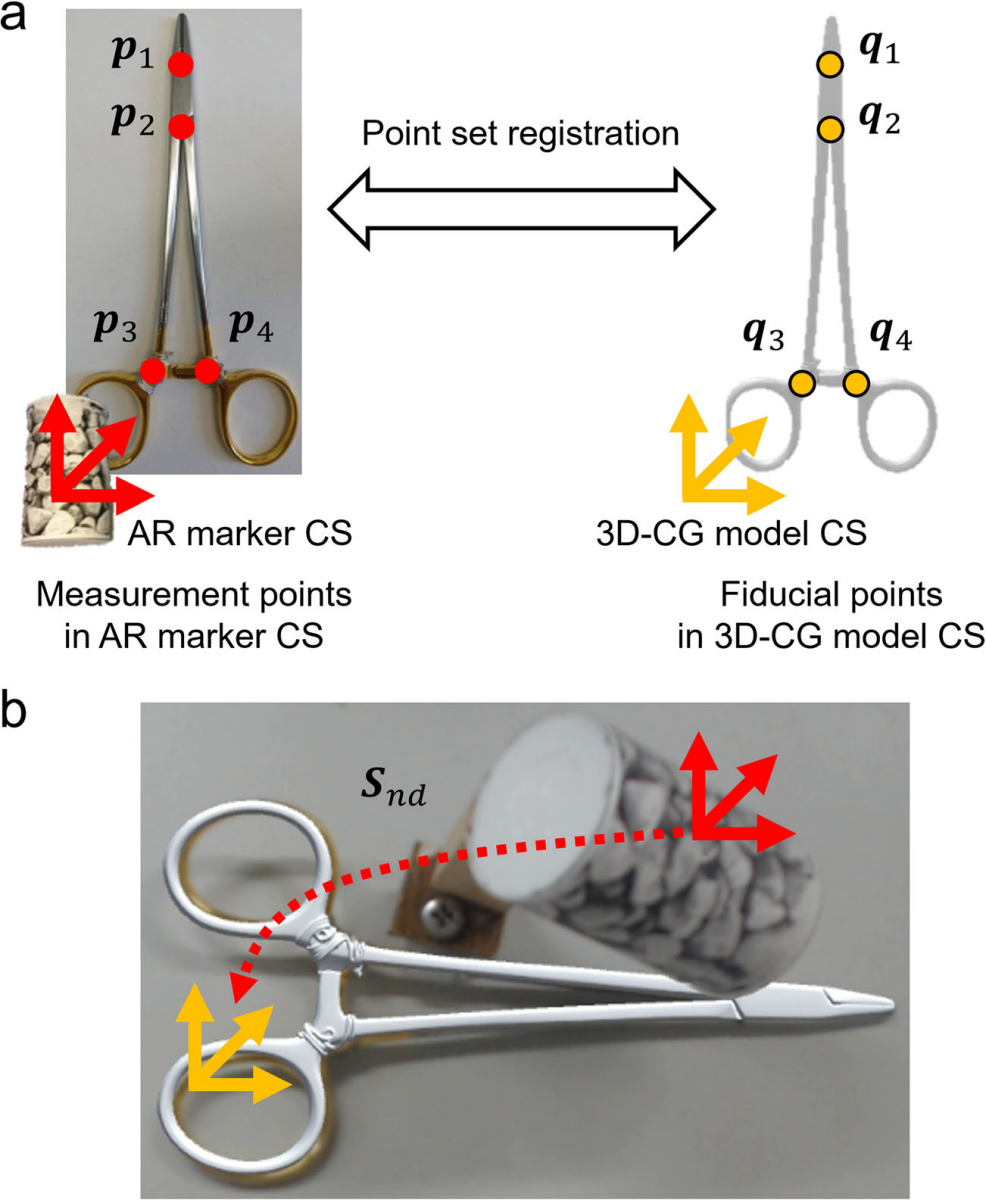
Point set registration for the needle driver. **a** Point set registration between measurement points (***p***_*i*_) on the actual needle driver and fiducial points (***q***_*i*_) on the 3D-CG model of the needle driver. **b** Coordinate transformation to display the 3D-CG model according to the AR marker

To replicate the motion of actual surgical instruments using AR technology, it is necessary to obtain the positional relationship between the AR marker and the actual surgical instrument. Based on the AR marker, a 3D-CG model of the surgical instrument is displayed in the same position and orientation as the actual surgical instrument. Therefore, the 3D-CG models were reconstructed by 3D scanning, and coordinate transformations between the AR marker coordinate system (CS) and the 3D-CG model CS of each surgical instrument were calculated using point set registration before motion capture. As shown in Fig. 2, using 4 points for the needle driver and 7 points for the forceps, point set registration was performed between the coordinates of measurement points on the actual surgical instrument in the attached AR marker CS (***p***_*i*_) measured by an AR probe and the coordinates of fiducial points on the 3D-CG model in the 3D-CG model CS (***q***_*i*_). The coordinate transformations ( ***S***_*nd*_ for needle driver, ***S***_*f*_ for forceps) were obtained respectively by the following equation and saved as text files.

$$ \boldsymbol{S}=\arg \underset{{\boldsymbol{S}}^{\prime }}{\min }{\sum}_{i=1}^n\left({\boldsymbol{p}}_i-{\boldsymbol{S}}^{\prime }{\boldsymbol{q}}_i\right) $$

**Fig. 3:**
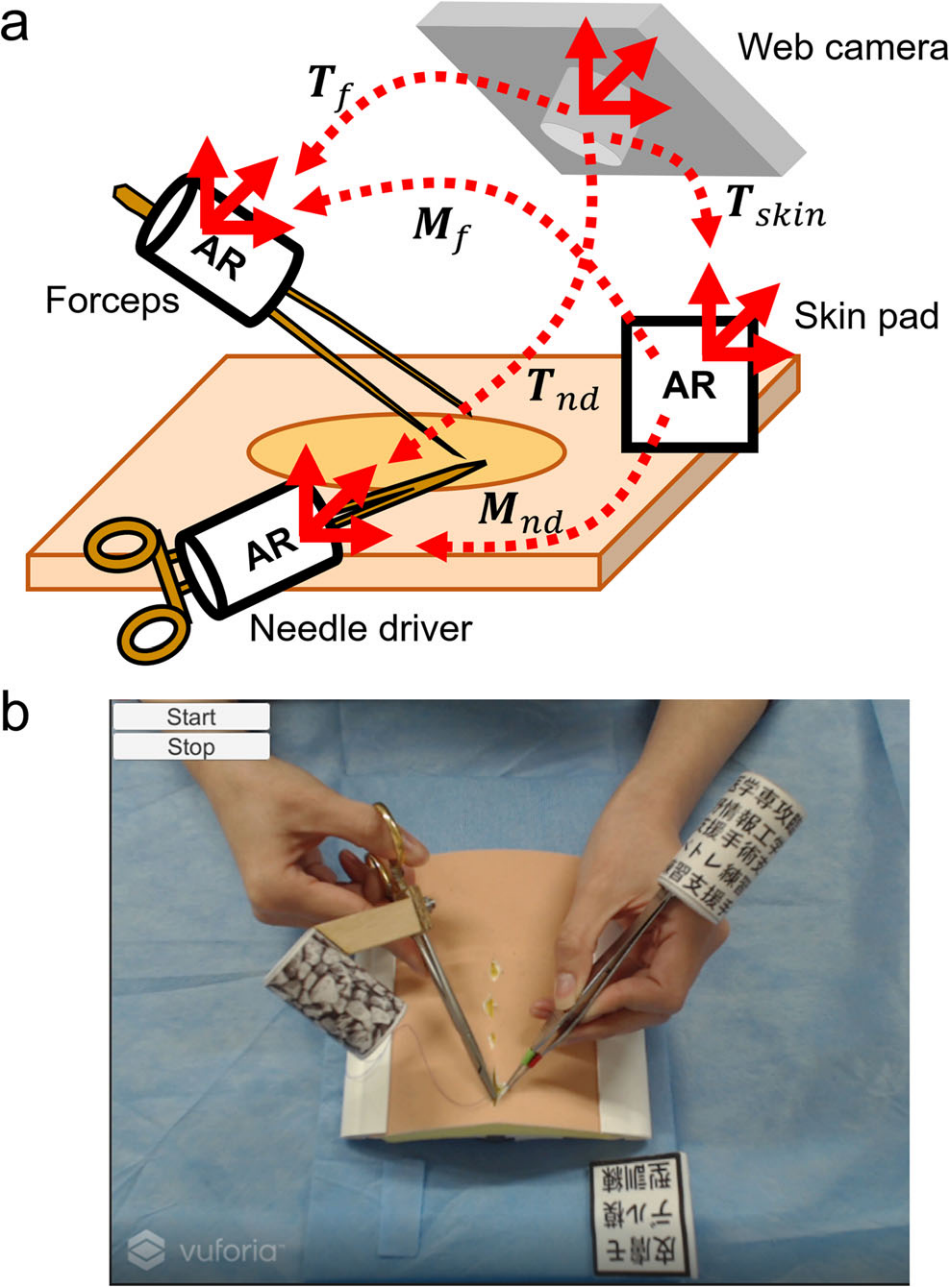
Motion capture system for surgical instruments. **a** Schematic representation of the motion capture system using AR markers and a web camera. **b** Screenshot during motion capture

When performing the procedure for capturing motion, users pressed the start button in the Unity editor, and the CS of each AR marker (***T***_*nd*_**,** ***T***_*f*_**,** ***T***_*skin*_) from the camera was tracked at 30 frames per second (fps) using our software (Fig. 3). Each AR marker CS on surgical instruments was transformed to the reference marker CS so that the data were replicated as captured on the surgical field regardless of the camera position. Relative CSs from the reference marker to the needle driver marker (***M***_*nd*_) and forceps marker (***M***_*f*_) were calculated and recorded for each frame. When users finished the procedure, they pressed the stop button, and the data were saved as text files.

**Fig. 4:**
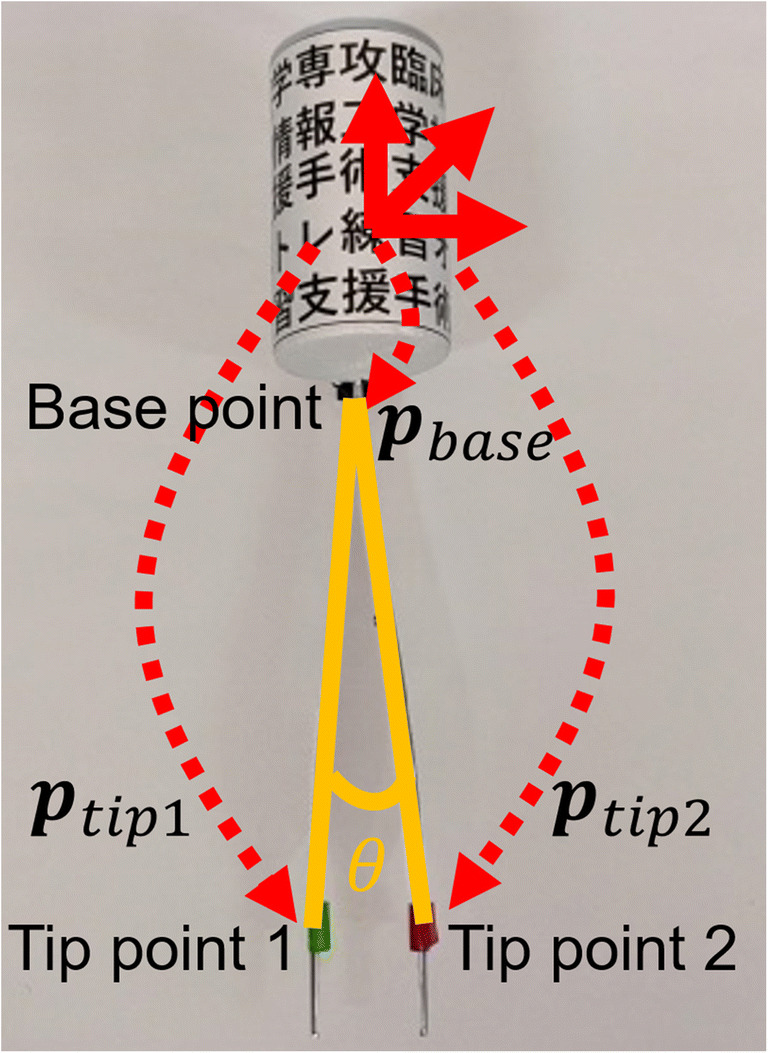
Calculation of the angle between tips from the base point of the forceps

The forceps opening angle (*θ*) was also recorded to replicate the open/closed condition of the forceps so that users who observe the replicated procedures can understand the correct place where the forceps should grasp the skin. Each tip of the forceps (Tip point 1 and Tip point 2) was marked and tracked from the camera. The angle was calculated using the position of the marks (***p***_*tip*1_, ***p***_*tip*2_) and the base point (***p***_*base*_) in the AR marker CS by the following equation for each frame, as described in Fig. 4, and saved simultaneously with the CSs of the surgical instruments (***M***_*nd*_, ***M***_*f*_).

$$ \theta ={\cos}^{-1}\left(\frac{{\boldsymbol{p}}_{tip1}-{\boldsymbol{p}}_{base}}{\left|{\boldsymbol{p}}_{tip1}-{\boldsymbol{p}}_{base}\right|}\bullet \frac{{\boldsymbol{p}}_{tip2}-{\boldsymbol{p}}_{base}}{\left|{\boldsymbol{p}}_{tip2}-{\boldsymbol{p}}_{base}\right|}\right) $$

### 3D replication system of procedures on the surgical field

In the replication system, the same skin pad used in the motion capture system was used. An AR application was developed for HoloLens 2 to track the reference marker and overlay 3D-CG models of surgical instruments on the skin pad. To replicate the open/closed state of the forceps, an animation was created using Blender ver. 2.79b (The Blender Foundation, Amsterdam, Netherlands) that changes the angle between the forceps’ tips in the 3D-CG model.

When overlaying information in AR applications, occlusion, the ability to hide 3D-CG objects located behind other objects, plays an essential role in providing the right spatial positional relationship [18]. A hand occlusion function was provided to maintain the relationship between hands and 3D-CG models in our application. HoloLens 2, in combination with MRTK, can track and visualize hands by hand mesh. The function was implemented by setting the hand mesh material as occlusion material prepared in MRTK for Unity 3D.

**Fig. 5:**
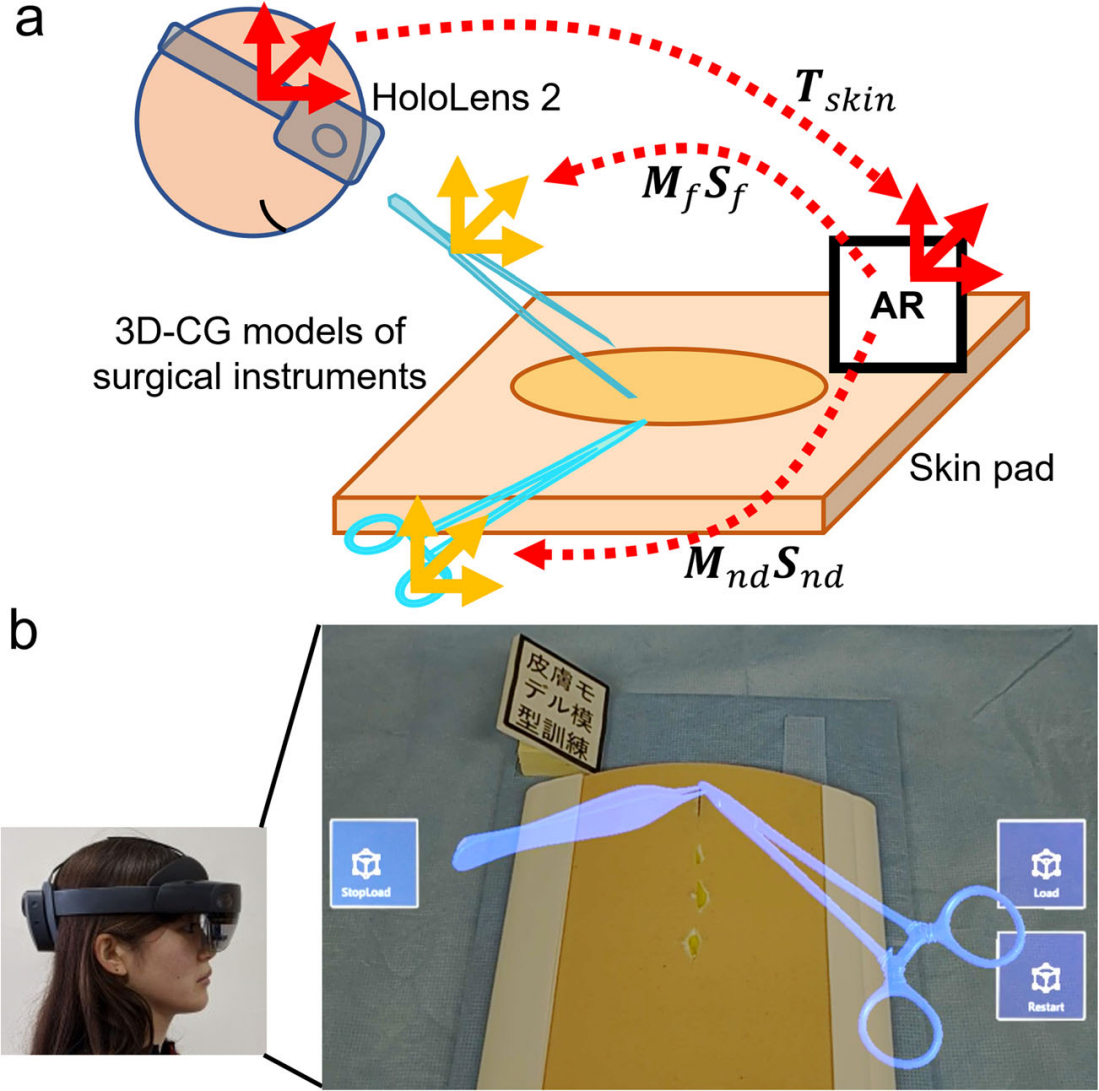
3D replication system of procedures on the surgical field. **a** Schematic representation of the replication system using HoloLens 2 and a skin pad. **b** Screenshot of HoloLens 2 during replication of the procedure. The brightness of all the screenshot images of HoloLens 2 is changed to 140% in PowerPoint to make them resemble the actual view from HoloLens 2, since the original image is darker than that which users see with their own eyes through HoloLens 2

When practicing with this system, users launched the application in HoloLens 2 and looked at the surgical field through HoloLens 2. The application has three buttons: a load button, stop button, and restart button. Once HoloLens 2 detected the reference marker, users pressed the load button. Then, text files saved in the motion capture system were loaded, and the position and orientation of each 3D-CG model of the surgical instruments were set to ***M***_*nd*_***S***_*nd*_ and ***M***_*f*_***S***_*f*_, respectively, for each frame (Fig. 5). The angle between the forceps’ tips was also set as recorded. Replication was repeated until the stop button was pressed and restarted from the beginning when the restart button was pressed.

## Experiments

### Surgical instruments’ tracking stability among different AR marker shapes

**Fig. 6:**
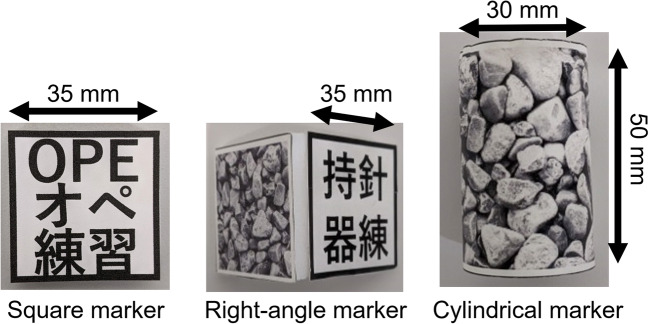
Candidate shapes of AR markers for tracking surgical instruments

Three types of AR markers were investigated for the needle driver: a cylindrical marker, a square marker, and a right-angle marker with two square markers glued at 90 degrees (Fig. 6). Square markers and cylindrical markers were prepared for the forceps. The cylindrical marker’s size was 30 mm in diameter and 50 mm in height, and the square marker size was 35 mm. AR markers were attached between the tip and handle for the needle driver and the top-end for the forceps.

Two experts performed subcuticular interrupted sutures five times for each shape, and both the total time in which AR markers were tracked and the procedure time were obtained in each suture. The tracked time to procedure time ratio was analyzed using one-way analysis of variance (ANOVA), followed by the Tukey-Kramer test when the ANOVA model’s findings were significant. Statistical analyses were conducted using Bell Curve for Excel (BellCurve, Tokyo, Japan). The *α* level (type I error) was set at 0.05.

### Training with the replicated procedure

The performance and usability of the replication system were evaluated.

An expert’s subcuticular interrupted suture procedure was recorded using the motion capture system and used in the replication system. Whether HoloLens 2 detected the reference marker and at what frame rate it visualized the procedure on the surgical field were examined. For usability evaluation, whether users could see the replicated procedure and the actual surgical instruments simultaneously and imitate the procedure by manipulating them were explored. Whether the 3D-CG models were occluded by the hands when users placed their hands in front of them was also investigated.

## Results

### Motion capture system for surgical instruments

A motion capture system of surgical instruments for subcuticular interrupted suture was developed using AR markers and a camera.

During the procedure, all markers were tracked from the camera simultaneously without interfering with each other. According to the experts, the markers did not interfere with the manipulation of surgical instruments or obstruct the line of sight to the surgical instruments’ tips, enabling them to perform the procedure as usual. The tracking data could be obtained at 30 fps regularly.

**Fig. 7:**
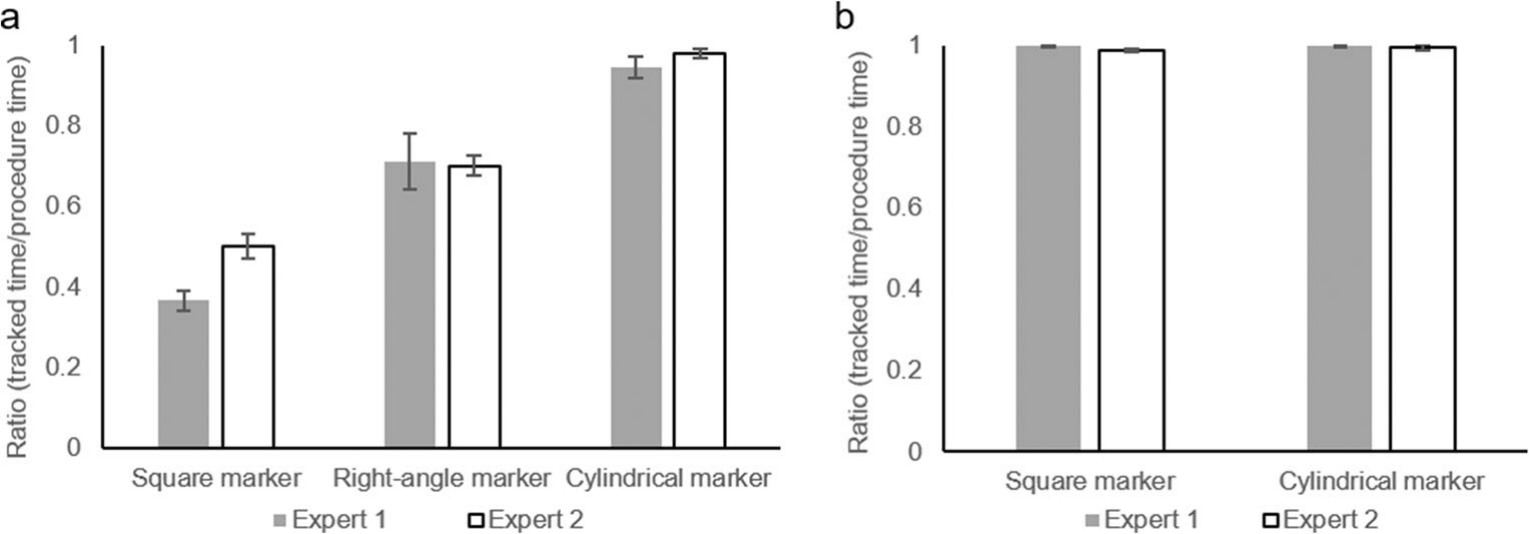
Surgical instruments’ tracking stability among candidate shapes of AR markers during procedures by two experts. The Y-axis shows the ratio of the tracked time of each AR marker to the procedure time. **a** Needle driver. **b** Forceps

The average and the standard error of the ratio of tracked time to procedure time were: expert 1 (0.37 ± 0.02 (Square marker); 0.71 ± 0.07 (Right-angle marker); 0.95 ± 0.03 (Cylindrical marker)); expert 2 (0.50 ± 0.03 (Square marker); 0.70 ± 0.03 (Right-angle marker); and 0.98 ± 0.01 (Cylindrical marker)) (Fig. 7a). ANOVA demonstrated a significant difference among the three AR markers for both experts (F (2, 12) = 32.9506, *p* < 0.001 for expert 1; F (2, 12) = 79.9424, *p* < 0.001 for expert 2), and the Tukey-Kramer test showed significant differences (*p* < 0.05) for all pairs of AR markers in both experts. The cylindrical AR marker could be tracked when the needle driver was rotated, whereas the square marker could not. The right-angle marker was also lost when the needle driver’s rotation switched the marker visible from the camera. For the forceps, both the square marker and the cylindrical marker were stable (1.00 ± 0.00 (Square marker); 0.99 ± 0.00 (Cylindrical marker) for expert 1, and 1.00 ± 0.00 (Square marker); 0.99 ± 0.01 (Cylindrical marker) for expert 2) (Fig. 7b). However, the CS estimation of the square marker was sometimes inaccurate when observed from the frontal direction, as shown in a previous study [19]. Therefore, cylindrical AR markers were selected for tracking both surgical instruments. Both tip points on the forceps could be tracked throughout the procedure. The angle between the tips was recorded without lacking data.

### 3D replication system of procedures on the surgical field

A 3D replication system of surgical procedures was developed using HoloLens 2.

As shown in Fig. 5, when a user saw the skin pad through HoloLens 2, HoloLens 2 tracked the reference marker and visualized the procedure captured by the motion capture system three-dimensionally according to the marker at around 20 fps. Consequently, the procedure was replicated in the same position as recorded, including the forceps tips angle, and users could observe it from their desired angle.

**Fig. 8:**
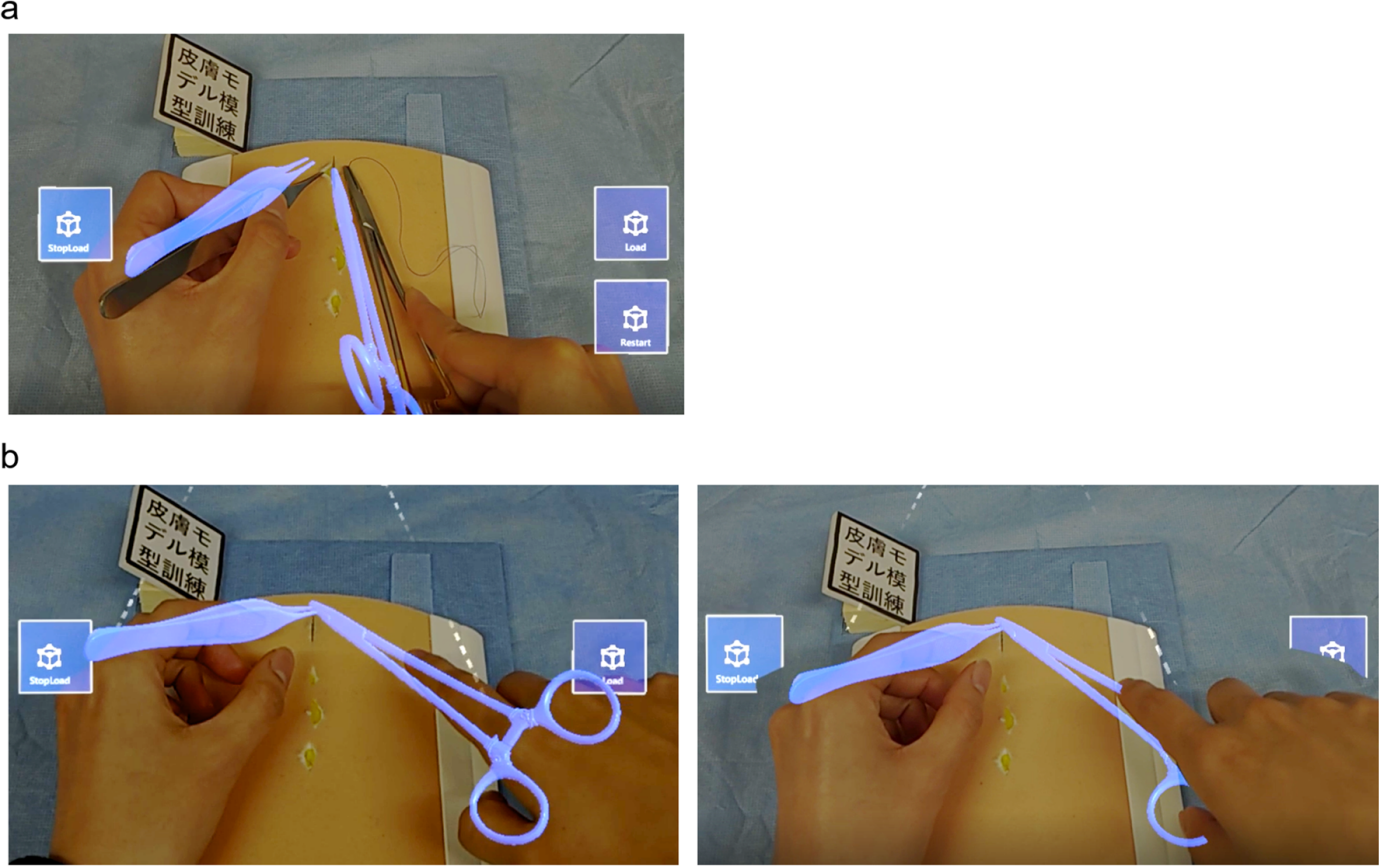
HoloLens 2 screenshot while practicing with the replication system. **a** Imitating the replicated procedure by manipulating the actual surgical instruments. **b** Occlusion of 3D-CG models by hands. Left: without hand occlusion. Right: with hand occlusion. A part of the 3D-CG model of the needle driver is occluded by the hand placed in front of the model

When users positioned themselves to suture in front of the skin pad, they could see the entire 3D-CG models of surgical instruments within a field of view (FOV). They could also see both 3D-CG models and the actual surgical instruments simultaneously and imitate the replicated procedure by manipulating the actual surgical instruments (Fig. 8a).

The displayed 3D-CG models were occluded by the user’s hands when their hands were placed in front of them (Fig. 8b). However, the frame rate dropped to about 10 fps due to the calculation of hand mesh occlusion, which reduced the smoothness of the procedure’s motion.

## Discussion

A novel suture training system for open surgery that provides 3D information of exemplary procedures during practice by combining AR technology with a low-fidelity simulator was developed.

Motion capture of surgical instruments can be done using electromagnetic, mechanical, or optical systems, and in any case, the systems must be minimal and nonobstructive [13]. Motion capture is more complicated in open surgery than in endoscopic surgery due to the greater DOF of surgical instruments’ motion [12, 13]. In the present study, using AR markers, it was possible to track the position and orientation of surgical instruments and the surgical field throughout the procedures without occlusion of AR markers or obstruction of the procedures. For AR markers’ size, the minimum size was 30 mm to be tracked from the camera placed 300 mm above the skin pad, since the Vuforia engine can detect a marker from distances of up to about ten times the marker size [20]. Simultaneously, AR markers should be sufficiently small not to interfere with the procedures. Therefore, the size used in the system was appropriate to meet these two requirements. For AR markers’ shape, cylindrical markers may be more suitable than square markers for tracking surgical instruments’ motion in open surgical procedures with high DOF, since they were tracked accurately even if the surgical instruments were rotated.

AR markers are a low-cost method widely used in AR applications for CS estimation from a camera [21]. By using AR markers, our motion capture system retains the advantages of low-fidelity simulators (i.e., portable, low cost, and does not require dedicated facilities), because it only needs a camera, AR marker-attached surgical instruments, and low-fidelity simulators.

The present replication system visualized the procedures performed by experts three-dimensionally at the same site as performed. This system allowed trainees to observe the procedures from their desired angle, even in the experts’ absence, which is not possible using conventional teaching materials. In addition, trainees could access the procedures without stopping practice because they could see both the overlayed procedures and the real surgical field simultaneously [15]. It is also possible to replicate the procedures in slow motion by varying the rate of data loading, allowing trainees to observe them closely for a better understanding. Therefore, once trainees asked their instructors to perform the procedure and recorded it with the motion capture system, they could practice it repeatedly with the replication system at any time using actual surgical instruments. We expect that the hand occlusion function will allow trainees to experience the procedure as if they were holding the 3D-CG models in their hands.

HoloLens 2 has a sufficient FOV to display the entire 3D-CG models of surgical instruments within its FOV, allowing trainees to grasp their position and orientation immediately and imitate the replicated procedures while manipulating the actual surgical instruments. This feature will likely help trainees efficiently understand the ideal procedures and recognize the differences between their procedures and experts’ procedures and what needs to be improved.

The procedure was replicated at around 20 fps without hand occlusion, which was slower than the original, but the procedure’s motion was smooth. However, hand occlusion reduced the smoothness due to the lower fps. Although the central processing unit in AR devices has been improving, it needs further improvement to render 3D-CG models and the hand mesh simultaneously without degrading the user experience.

Since this training system can be built by introducing AR markers and AR devices to existing low-fidelity simulators, the system will also apply to various suture practices in open surgery, such as bowel anastomosis, using low-fidelity simulators. In addition, there are commercially available devices that can track users’ fingers, and HoloLens 2 itself tracks users’ fingers. Therefore, by combining these devices with the system, it will also be able to replicate the motion of a surgeon’s fingers, such as when tying knots.

Although the present system provides a new learning experience, there are some limitations. First, the accuracy of the replicated procedures to the original procedure was not validated. In addition, an objective evaluation of the training effectiveness of this system is still lacking. Further validation is needed to confirm the reliability and effectiveness of the system. Needles were not visualized, because the positional relationship between needles and needle drivers is fixed during the procedure. Visualizing needles will be necessary to practice other procedures in which the positional relationship has to be changed.

Although the AR markers did not obstruct this study’s procedures, they may still be too bulky to be applied to other procedures. Technological advances in marker recognition will make the marker size smaller and solve this problem.

## Conclusion

An inexpensive motion capture system for open surgery was developed by evaluating AR markers’ shapes, and a suture training system that allows trainees to practice with replicated exemplary procedures using HoloLens 2 was proposed. This training system will contribute to helping trainees learn surgical skills by themselves without an instructor.

## Declarations

### Code availability

The code used for the development of the application is available from the corresponding author on reasonable request.
